# Supplementary material for: Linguistic metaconcepts can improve grammatical understanding in L1 education evidence from a Dutch quasi-experimental study
Source: PLoS One. 2022 Feb 3;17(2):e0263123. doi: 10.1371/journal.pone.0263123 (PMC8812858; doi:10.1371/journal.pone.0263123)
Supplement: S1 File — (DOCX) [file pone.0263123.s001.docx]

**Appendix A – detailed information on the intervention (4 x 50 minutes)**

| Lesson | Assignments | Theory covered in the lesson | Dominant design principles |
| --- | --- | --- | --- |
| 1 | 1. Exercise with plasticized cards containing various word classes. Students have to make three equal groups from these (pairs). 2. Exercise with plasticized cards containing verbs and noun phrases meant to discover valency patterns (groups of 4). Assignment has been described in some detail in Van Rijt (2020). 3. Valency exercise in which students try to assess how many roles the given verbs serve out, with follow-up assignments in which the students are prompted to draw conclusions based on the previous exercises. (Individually). One of these is **Key Assignment 1**. | After cards exercise (2): theory about *valency*, relating the concept to concepts from secondary school grammar: *verb*, *subject*, *direct object*, *indirect object* and *preposition*. | 1. All (particularly: dealing with grammatical uncertainty, since some cards might be placed in two groups) 2. All 3. All |
| 2 | 1. Valency exercise in which students try to assess how many roles the given verbs serve out (repetition from lesson 1, to activate prior knowledge). (Pairs). 2. Odd one-out exercise with sets of verbs (pairs) 3. Parsing exercise based on valency (individually) 4. Exercise about cases of reduced valency (valency reduction) and subsequent follow-up assignments (pairs) 5. Communicative assignment about valency reduction (individually, **Key Assignment 2**). 6. Exercise aimed at making the students discover that valency can sometimes also be increased (valency addition), and short follow-up assignments meant to strengthen their discovery | Theory covering the fact that *not all verbs serve out roles*; only *main verbs*. The theory thus links valency to main verbs, and distinguishes main verbs from *auxiliary verbs* and *copular verbs*. | 1. Linking metaconcepts to traditional concepts; dealing with uncertainty; stimulating talk 2. Dealing with uncertainty, inductive learning, stimulating talk 3. Linking metaconcepts to traditional concepts 4. Guided inductive assignment, stimulating talk 5. Guided inductive assignment 6. Guided inductive assignment, linking metaconcepts to traditional concepts |
| 3 | 1. Exercise meant to let students discover that some verbs are accompanied by fixed prepositions (prepositional objects). (pairs) 2. Exercise meant to let students discover that prepositions from prepositional objects can readily be modified (see theory).(pairs) 3. Reasoning excercise (groups of four) in which students are presented with a grammatical problem, for which two possible solutions are presented. They are also presented with arguments, and they have to decide a.) whether the arguments are relevant and true or not; and b.) which of the arguments relates to which possible solution. They then need to arrive at a joint decision about which solution is best based on the arguments given. | Theory covering the *prepositional object*, and linking this object to the metaconcept of *valency*. The theory also deals with linguistic tests to establish whether or not we are dealing with prepositional objects, by drawing on the modification of the preposition (if it cannot be modified, it is a prepositional object, otherwise, we are dealing with adverbials). | 1. Guided inductive assignment, stimulating talk 2. Guided inductive assignment 3. Dealing with uncertainty; linking metaconcepts to traditional concepts; stimulating exploratory talk |
| 4 | 1. Assignment: formulate the difference between objects and adverbials based on what you have learned in the previous lessons. (individually) 2. Odd one-out: which of the following verbs is the odd one-out, and why? (pairs and individual processing) **Key Assignment 3** 3. Odd one-out: which of the underlined phrases is the odd one-out, and why? (pairs and individual processing) **Key Assignment 3** 4. Reasoning exercise (see lesson 3). 5. Reasoning exercise (see lesson 3). | N/A | 1. Linking metaconcepts to traditional concepts. 2. Dealing with uncertainty, linking metaconcepts to traditional concepts, stimulating talk 3. Dealing with uncertainty, linking metaconcepts to traditional concepts, stimulating talk 4. Dealing with uncertainty; linking metaconcepts to traditional concepts; stimulating exploratory talk 5. Dealing with uncertainty; linking metaconcepts to traditional concepts; stimulating exploratory talk |

*Description of the intervention in more detail. Key Assignments for qualitative analyses are marked in bold face.*

**Appendix B – three versions of the Test for Grammatical Understanding (TGU) (translated from Dutch by the authors*).***The alternatives are ranked from full understanding (A), to partial understanding (B), blind concept use (C) and no concept use (D).*

| Question | TGU Version 1 | TGU Version 2 | TGU Version 3 |
| --- | --- | --- | --- |
| 1 | Why can you leave out ‘a book’ in the sentence ‘Jan reads a book’, whereas you cannot do this in ‘Jan tears up a book’?   1. The verb ‘to tear up’ selects a mandatory direct object. 2. The direct object ‘a book’ can sometimes be left out, and sometimes not 3. The past participle sounds similar to the finite verb 4. You can’t say what you tear up without saying what you are tearing up | Why can you leave out ‘a sandwich’ in the sentence ‘Astrid has eaten a sandwich’, whereas you cannot do this in ‘Astrid has made a sandwich’?   1. The verb ‘to make’ selects a mandatory direct object 2. The direct object ‘a sandwich’ can sometimes be left out, and sometimes not 3. The auxiliary verb ‘has’ sometimes requests a direct object 4. You can’t say what you make without saying what you are making. | Why can you leave out ‘his nephew’in the sentence ‘Peter seems to have struck his nephew’, whereas you can’t do this in ‘Peter seems to have acknowledged his nephew’?   1. The verb ‘to acknowledge’ selects a mandatory direct object 2. The direct object ‘his nephew’ can sometimes be left out, and sometimes not 3. The auxiliary verb ‘seem’ sometimes requests a direct object 4. You can’t say that you have acknowledged someone without saying whom |
| 2 | Your Dutch language teachers asks you to construct a grammatical sentence using the pattern below, with parts of speech 1-6. What can you say about this sentence in advance?   \| 1 \| 2 \| 3 \| 4 \| 5 \| 6 \| \| --- \| --- \| --- \| --- \| --- \| --- \| \| Dir. object \| Fin. verb \| Subj. \| Ind. object \| Adv. \| Adv. \|  1. (2) can have the verb ‘to blame’, because this verb asks for a direct and an indirect object 2. The sentence is unusual because it has the direct object in sentence first position rather than the subject 3. The sentence is impossible, because there can’t be two adverbials in one sentence 4. The sentence consists of minimally six words, because there are six spaces to use | Your Dutch language teachers asks you to construct a grammatical sentence using the pattern below, with parts of speech 1-6. What can you say about this sentence in advance?   \| 1 \| 2 \| 3 \| 4 \| 5 \| 6 \| \| --- \| --- \| --- \| --- \| --- \| --- \| \| Ind. object \| Fin. verb \| Subj. \| Dir. object \| Adv. \| Adv. \|  1. (2) can have the verb ‘to award’, because that verb asks for a direct and an indirect object 2. The sentence is unusual because it has the indirect object in sentence first position rather than the subject 3. The sentence is impossible, because there can’t be two adverbials in one sentence 4. The sentence consists of minimally six words, because there are six spaces to use | Your Dutch language teachers asks you to construct a grammatical sentence using the pattern below, with parts of speech 1-5. What can you say about this sentence in advance?   \| 1 \| 2 \| 3 \| 4 \| 5 \| \| --- \| --- \| --- \| --- \| --- \| \| Fin. verb \| Subj. \| Adv. \| Ind. Obj. \| Dir. Obj. \|  1. (1) can have the verb ‘to offer’, because that verb asks for a direct and an indirect object 2. The sentence is unusual because it has the finite verb in sentence first position rather than the subject 3. The sentence is impossible, because there can never be an adverbial after the subject 4. It is highly likely that there will be a person on (2). |
| 3 | Some sentences have more than one meaning, such as ‘Louise has seen the men with binoculars’. What causes the difference in meaning?   1. ‘With binoculars’ can get it’s meaning from the verb, or from the connection with the noun 2. You can place both ‘the men’ and ‘the men with binoculars’ before the finite verb 3. The preposition ‘with’ is ambiguous in this sentence; it can either mean ‘together with’ or ‘with the help of’ 4. It depends on who hold the binoculars in reality, Louise or the men | Some sentences have more than one meaning, such as the headline ‘Suspect of liquidation released on A73’. What causes the difference in meaning?*   1. ‘On A73’ can get it’s meaning from the verb, or from the connection with the noun 2. You can place both ‘A73’ and ‘Suspect of liquidation on A73’ before the finite verb 3. The preposition ‘on’ is ambiguous in this sentence; it can either mean ‘on top of’ or ‘at the altitude of’ 4. It depends whether the liquidation or the release has happened on the A73 | Some sentences have more than one meaning, such as the headline ‘Prosecutor wants that man with hammer is treated’. What causes the difference in meaning?*   1. ‘with hammer’ can get it’s meaning from the verb or from the connection with the noun 2. You can place both ‘man’ and ‘man with hammer’ before the finite verb 3. The preposition ‘with’ is ambiguous in this sentence; it can either mean ‘together with’ or ‘with the help of’ 4. It depends on whether the prosecutor wants the man to be tortured or treated |
| 4 | The sentence *Ik wacht op het perron* (I am waiting on the platform) resembles ‘*Ik wacht op beter weer* (I am waiting for better weather). But you can say ‘Ik wacht boven op het perron’ (I am waiting on top of the platform), but not ‘Ik wacht boven op beter weer’ (I am waiting on top for better weather’). Why is that?*   1. In ‘I am waiting for better weather’, the preposition has no meaning of place 2. The preposition *op* can sometimes not be modified by an adverb 3. The verb *to wait* requires a fixed preposition 4. You can stand on a plaform, but you can’t stand on the weather | The sentence ‘De leraar verschuilt zich achter die struik’ (the teacher hides behind that bush) resembles ‘De leraar verschuilt zich achter zijn verantwoordelijkheid’ (the teacher hides behind his responsibility). But you can say ‘De leraar verschuilt zich *dicht* achter die struik’, but not ‘De leraar verschuilt zich *dicht* achter zijn verantwoordelijkheid’. How come?*   1. In ‘De leraar verschuilt zich achter zijn verantwoordelijkheid’ the preposition has no meaning of place 2. The preposition *achter* can sometimes not be modified by an adverb 3. The verb *verschuilen* requires a fixed preposition 4. You can stand behind a bush, but you can’t stand behind responsibility | The sentence ‘De buurvrouw staat stil bij de vensterbank’ (the neightbour stands still at the windowsill) resembles ‘De buurvrouw staat stil bij onrechtvaardigheid’ (the neightbour pays attention to injustice). But you can say ‘De buurvrouw staat stil *dicht* bij de vensterbank’, but not ‘De buurvrouw staat stil *dicht* bij onrechtvaardigheid’. How come?*   1. In ‘De buurvrouw staat stil bij onrechtvaardigheid’ the preposition has no meaning of place 2. The preposition *bij* can sometimes not be modified by an adverb 3. The verb *stilstaan* requires a fixed preposition 4. You can stand near the windowstill, but you can’t stand near injustice |
| 5 | The following sentence is in the school paper: ‘Docenten rekenen op een nieuw schoolbord’ (teachers count on / calculate on a new blackboard). The maths teacher thinks the sentence is about a blackboard that he can calculate on, but the principal thinks the sentence means that teachers are expecting the arrival of new blackboards. How come they read this sentence differently?*   1. The preposition *op* can indicate place, but it can also be meaningless combined with certain verbs 2. You can take ‘op een schoolbord’ literally or figuratively here 3. The present tense steers the meaning in the direction of the future 4. Math teachers only think about calculations, and principals only about money | A news item has as its headline ‘Klimaatdemonstranten waarschuwen voor witte huis’ (*Climate protesters warn for/in front of White House*). One reader expects the article to describe protests that have occurred in front of the White House, whereas another expects the protesters to warn against the *policy* of the White House. How come they have different expectations?*   1. The preposition *voor* indicate place, but it can also be meaningless 2. You can read ‘voor Witte huis’ in a literal and in a figurative meaning here 3. The agreement between subject and finite verb indicates two meanings 4. Protesting near the White House happens a lot, and for many reasons | A news item has as its headline ‘Bedrijfsleiders verschuilen zich achter Nederlandse regering’ (corporate management hides behind Dutch government). One reader thinks that the corporate managers are literally standing behind the Dutch prime minister, whereas the other reads that managers don’t take their responsibility. How come they read this differently?*   1. The preposition *achter* can indicate place, but it can also be meaningless 2. You can read ‘achter Nederlandse regering’ in a literal and in a figurative meaning here 3. The reflexive pronoun *zich* causes the sentence to have two meanings 4. One reader simply has a different sense of imagination than the other |
| 6 | Review the following two sentences:   1. De studenten *zaten in* de collegebanken. (The students sat in the lecture desks) 2. De studenten *verdiepten zich in* de literatuur. (The students studied the literature)   In sentence (1), you can replace *in* with *onder*. That will then mean something different, but the replacement is possible. In (2) this is not possible. ‘De studenten verdiepten zich onder de literatuur’ is an impossible sentence. How come?*   1. In the case of objects, the verb determines the properties of the preposition, but not in the case of adverbials 2. *In de collegebanken* is an adverbial, and *in de literatuur* is an object of the verb 3. *Verdiepen* is a reflexive verb, always requiring *zich* 4. *Verdiepen* is all about diving into something, because it is about the depth | Review the following two sentences:   1. De docenten zullen *ingaan op* klimaatverandering. (The teachers will talk about climate change) 2. De docenten mogen *leunen op* tafels. (The teachers are allowed to lean on tables)   In sentence (2), you can replace ‘op’ with ‘on top of’, but that same replacement is impossible in (1). ‘De docenten zillen ingaan *boven op* klimaatverandering’ is an ingrammatical sentence. How come?*   1. In the case of objects, the verb determines the properties of the preposition, but not in the case of adverbials 2. *Op tafels* is an adverbial and *op klimaatverandering* is an object of the verb 3. The auxiliary verb *zullen* demands a different preposition than *mogen* 4. Leaning is always literally on top of something, which is not the case for *ingaan* | Review the following two sentences:   1. De leerlingen *verlangen naar* de kerstvakantie. (The students long for the christmas holiday) 2. De leerlingen *rennen naar* de schoolkantine. (The students run towards the cafeteria)   In (2) you can replace *naar* by *richting*. That will then mean something a little different, but the replacement is possible. In sentence (1), this is not possible. ‘De studenten verlangen richting de kerstvakantie’ is an impossible sentence. How come?*   1. In the case of objects, the verb determines the properties of the preposition, but not in the case of adverbials 2. *Naar de schoolkantine* is an adverbial, and *naar de kerstvakantie* is an object of the verb 3. *Naar de schoolkantine* is a mandatory adverbial in sentence (2) 4. You always run towards a destination, of in a certain direction |
| 7 | Why can you say ‘Ik weet wie het gedaan heeft’ (I know who did it), but not ‘Ik denk wie het gedaan heeft’ (I think who did it)?   1. Because the verb *denken* (to think) does not select a question phrase as its direct object 2. Because you can’t get an interrogative pronoun in the second sentence 3. Because the predicate at the end is in the present perfect 4. Because you are not completely sure about something if you are thinking about it | Why can you say ‘Ik begrijp wie het gedaan heeft’ (I understand who did it) but not ‘Ik verwacht wie het gedaan heeft’ (I expect who did it)?   1. Because the verb *verwachten* (to expect) does not select a question phrase as its direct object 2. Because you can’t get an interrogative pronoun in the second sentence 3. Because the predicate at the end is in the present perfect 4. Because you cannot be completely sure about something if you are expecting something | Why can’t you say ‘Ik denk of het gaat regenen’ (I think whether it will rain) but you can say ‘Ik denk dat het gaat regenen’ (I think it will rain)?   1. Because the verb *denken* (to think) does not select an *of*-sentence as its direct object 2. Because you cannot get *of* as a subordinate conjunction in the first sentence 3. Because the auxiliary verb *gaan* expresses future tense, which makes you unsure 4. Because you are not completely sure about something if you are thinking about it |
| 8 | Why is ‘De stoel bungeejumpt’ (the chair bungeejumps) not a normal sentence?   1. The verb *bungeejumpen* imposes restrictions on the meaning of the subject 2. *De stoel* cannot be the subject of the verb *bungeejumpen* 3. The verb *bungeejumpen* demands a mandatory direct object 4. You don’t ever see chairs bungeejumping in real life, it is impossible | Why is ‘De laptop wandelt’ (the laptop walks) not a normal sentence?   1. The verb *wandelen* (to walk) imposes restrictions on the meaning of the subject 2. *De laptop* cannot be the subject of the verb *wandelen* 3. The verb *wandelen* demands a mandatory direct object 4. You don’t ever see laptops walking around in real life, it is impossible | Why is ‘De tafel rent’ (the table runs) not a normal sentence?   1. The verb *rennen* (to run) imposes restrictions on the meaning of the subject 2. *De tafel* cannot be the subject of the verb *rennen* 3. The verb *rennen* demands a mandatory direct object 4. You don’t ever see tables running in real life, it is impossible |
| 9 | Why is the sentence ‘Mijn opa rookt altijd een lantaarnpaal’(My grandfather always smokes a lamppost) not a normal sentence?   1. The verb *roken* (to smoke) imposes restrictions on the meaning of the direct object 2. A lamppost’ cannot be the direct object of the main verb *roken* 3. The verb *roken* selects a mandatory direct object in normal sentences 4. In a normal world it is hard to imagine that a lamppost is smoked | Why is the sentence ‘De minister-president leest de toiletborstel’ (the prime minister reads the toilet brush) not a normal sentence?   1. The verb *lezen* (to read) imposes restrictions on the meaning of the direct object 2. *De toiletborstel* cannot be the direct object of the main verb *lezen* 3. The verb *lezen* selects a mandatory direct object in normal sentences 4. Under normal circumstances you cannot read a toiletbrush | Why is the sentence ‘Ik vertel een verhaal aan de theelepel’ (I am telling a story to the teaspoon) not a normal sentence?   1. The verb *vertellen* imposes restrictions on the meaning of the indirect object 2. *Aan de theelepel* cannot be the indirect object of the verb *vertellen* 3. The verb *vertellen* selects a mandatory indirect object in normal sentences 4. In a normal word talking to teaspoons is pretty odd |
| 10 | The following two sentences appear to be structured in the same way:   1. De directeur heeft *aan de conciërge* een sleutel overhandigd. (The director has given a key to the janitor) 2. De directeur heeft *aan de conciërge* een gedachte gewijd. (The director has devoted a thought to the janitor).   Yet, in (1), *aan de conciërge* is being called an indirect object, but not in (2). Why not?*   1. Because the preposition is not mandatory in the first sentence, but it is in the second 2. Because the verb *wijden* cannot be accompanied by an indirect object 3. Because the direct object in sentence (2) is used figuratively 4. Because you can given something to someone, but you can’t devote something to someone | The following two sentences appear to be structured in the same way:   1. Alex heeft aan zijn hond geen aandacht besteed. (Alex has not paid attention to his dog.) 2. Alex heeft aan zijn hond geen voer gegund. (Alex has not allowed his dog any food.)   Yet, *aan de hond* is being called an indirect object in (2), but not in (1). Why not?*   1. Because the preposition is not mandatory in the first sentence, but it is in the second 2. Because the verb *besteden* cannot be accompanied by an indirect object 3. Because the direct object in sentence (1) is used figuratively 4. Because you can allow something to someone, but you can’t pay something attention to someone | The following two sentences appear to be structured in the same way:   1. De rechter heeft aan de advocaat geen boodschap gehad. (The judge did not care about the laywer). 2. De rechter heeft aan de advocaat een boodschap bezorgd. (The judge has delivered a message to the laywer).   Yet, *aan de advocaat* is considered an indirect object in (2), but not in (1). Why not?*   1. Because the preposition is not mandatory in the second sentence, but it is in the first 2. Because the verb *hebben* cannot be accompanied by an indirect object 3. Because the direct object in sentence (1) is used figuratively 4. Because you can deliver something to someone, but you can’t have something to someone |
| 11 | The sentence ‘Zij stierf een zachte dood’ (she died a soft death) deals with a specific way of dying. Why is that?   1. An unusual direct object often evokes specific aspects of meaning 2. The verb *sterven* cannot normally evoke a direct object 3. You cannot make this sentence passive (‘A soft death was died by her’) 4. ‘To die a soft death’ is redundant: if you die, you’re dead | The sentence ‘Marie sliep een onrustige slaap’ (Marie slept a resless sleep) deals with a specific way of sleeping. Why is that?   1. An unusual direct object often evokes specific aspects of meaning 2. The verb *slapen* cannot normally evoke a direct object 3. You cannot make this sentence passive (‘A resless sleep was slept by her’) 4. You can sleep in many different ways, both restless and calm | The sentence ‘Jan praat onzin’ (Jan talks nonsense) deals with a specific way of talking. Why is that?   1. An unusual direct object often evokes specific aspects of meaning 2. The verb *praten* cannot normally evoke a direct object 3. You cannot make this sentence passive (‘Nonsense is being talked by Jan’) 4. People can say meaningful and nonsensical things while talking |
| 12 | At Mr. Coumans’ house, sometimes young people come to the door to collect for the local football club. He always says something like 'I already give to the volleyball club'. Although he does not say this literally, it is clear that he means that he does not wish to give *money* to the football club. Why is this clear?*   1. Leaving out the direct object leads to a slightly different meaning, namely ‘to donate’ 2. In the sentence uttered by Mr Coumans, the direct object has been left out 3. The sentence contains an adverbial *al*, which expresses that he is out of budget 4. In this situation, it is evident that it is about giving money | Two people are gossiping about their neightbour. During the gossip, one of them says: ‘I have heared it too. Stefan drinks. Although it is not said literally, the statement clearly revolves around drinking *alcohol*. How come this is clear?*   1. Leaving out the direct object leads to a slightly different meaning, expressing addiction 2. In the sentence from the example, ‘Stefan drinks’, the direct object has been left out. 3. The finite verb is in the present tense, so it is still currently happening. 4. You can deduce from the context that something serious is intended, and that drinking is not about orange juice | At Mr. Postma, companies sometimes come by the door hoping that he will take a subscription for something. To avoid having to listen to such companies for too long, Mr. Postma usually says something like ‘Ik sluit niet af aan de deur’(I don’t buy at the door). Although he does not say this literally, it is clear that he means he does not want to buy *a subscription*. How come this is clear?*   1. Leaving out the direct object leads to a slightly different meaning, namely ‘to subscribe’ 2. In the sentence used by Mr. Postma, the direct object has been left out 3. The adverbial *aan de deur* evokes thoughts about salesmen 4. In this situation, it is evident that it is about not buying a subscription |

* *Note Due to translations, several of the questions make less sense in English. These questions have been marked with an asterisk(*), and some Dutch translations have been given in those cases to facilitate understanding. Differences in translation might also have changed the length of the questions compared to each other. In the original version, all questions and responses were very similar in length. All questions have been carefully composed by two Dutch linguists (both authors of the present paper).*

**Appendix C – Key Assignments from the intervention for qualitative analysis and examples of adequate responses**

*Key Assignment 1 (lesson 1)*

Explain in your own words, based on what you have just learned, how it is possible that one sentence does contain a direct or indirect object, whereas another sentence does not.

*Adequate response*
An adequate response to this question would for example relate the occurence of objects to the verb, and say that depending on the roles served out by the verb, there can or cannot be objects in the sentence. For example, the verb *to walk* only requires a subject; the verb *to read* requires a subject and a direct object, and the verb *to give* additionally requires an indirect object.

*Key Assignment 2 (Lesson 2)*

1. Look at the image of Giro 555 (cooperating aid organizations) below. Something striking in the field of valency is on the image. What is that?
2. Do you find it useful that the advertisers deal with the roles of a verb in this way? Explain your answer. Describe the effect of this use of valency on how the poster will be perceived.

*Adequate response*Students should observe that in one or more sentences on the poster (‘Give for food!’, ‘Give now!’) a role of the verb (the direct object) has been left out. This was probably done deliberately, as to emphasize the giving itself rather than what to give, since this can be readily inferred. In addition, all sentences are in the imperative, which means that the subject is dropped (although this is not the key valency issue of the poster).

*Key Assignment 3 (lesson 4)*

1. Which of the following underlined constituents is the odd one-out?
   1. Jan ziet elke dag vele politici.
      [Jan sees many politicians every day]
   2. Oma bakt voor ons vandaag weer een appeltaart.
      [Grandmother bakes us another apple pie again today]
   3. De boer rijdt op een tractor over het terrein.

[The farmer drives over the terrain on a tractor]

X is the odd one-out, because the other two …

1. Which verb is the odd one-out?
   1. Groeien
      [To grow]
   2. Krijgen

[To receive]

- 1. Roken

[To smoke]

X is the odd one-out, because the other two …

There are many adequate responses to these questions.

[Supplementary file: link to data used in this study].
