# Supplementary material for: Linguistic metaconcepts can improve grammatical understanding in L1 education evidence from a Dutch quasi-experimental study
Source: PLoS One. 2022 Feb 3;17(2):e0263123. doi: 10.1371/journal.pone.0263123 (PMC8812858; doi:10.1371/journal.pone.0263123)
Supplement: Attachment — Submitted filename: Response to the reviewers.docx [file pone.0263123.s002.docx]

**Response to the reviewers**

**Dear editor and dear reviewers,

Thank you for your positive evaluation of our manuscript. We have carefully considered your comments and suggestions and we feel that the manuscript has improved as a result. We hope that you feel the same way and that the manuscript is acceptable to you in this form. Below, we will outline how we have taken up every comment. In one of the uploaded files, all changes have been marked yellow.**

**Kind regards, on behalf of the other authors,**

**Dr. Jimmy H.M. van Rijt
Tilburg University**

**Response table**

| **Reviewer comment** | **Authors’ response** |
| --- | --- |
| **R1** |  |
| This is a very interesting and relevant study. The research problem is very well-motivated, the objectives are stated clearly, the methodological design seems adequate and robust, the number of participants recruited for the experiment is also impressive. Furthermore, the results provide encouraging evidence concerning the positive impact of meta conceptual grammar teaching on grammatical understanding. In general, I feel like the paper has merit and should be published once some adjustments/corrections are made and some points are more appropriately addressed. The paper is very well-written; however, some textual issues deserve attention. I would recommend a major revision of punctuation, for instance. | Thank you for this evaluation – we are happy that you feel this way!  We have followed up on practically all of the suggestions you have done; if not, we will explain why. Rest assured that the manuscript has been thoroughly checked for punctuation 😊 |
| Page 5: I feel like these two parts within the paragraphs should be better articulated, perhaps changing their order?: "This type of grammar teaching, focusing more on the mechanic articulation [...]" and "On the other hand, contextualized forms of grammar teaching [...]". | We agree; we have linked the sentences more in the following way: “On the other hand, less traditional and more contextualized forms of grammar teaching, such as those informed by Systemic Functional Linguistics …”, so it is clear that functional approaches are contrasted with traditional forms of grammar teaching here. |
| Page 12: You should not assume all your readers are aware of what "valency" is. Please, provide an explicit definition and, if possible, some examples. | We agree. A definition was already given earlier (p. 6 in both the new and original ms), but we have included a few examples there to clarify the metaconcept:  For example, different verbs have different slots available for subjects and objects: the verb to shiver only has a slot available for a subject (e.g., ‘HE shivers’, not ‘HE shivers SOMETHING’), whereas the verb to read has a slot available for both a subject and a direct object (e.g., SHE reads SOMETHING). Some verbs, such as the verb to give, even have three slots, namely for a subject, a direct and an indirect object (e.g., THE WIZARD gave THE CAT SOME FOOD).  The definition itself has been sharpened as well. |
| Page 13: "Teachers were told that they could  deviate from the specifics of the intervention to a limited degree if the situation called for it [...]": Okay, did they deviate much? I suggest you anticipate something about it or just say that it will be described further. | We added the following sentence to clarify:  Teachers did not report any major deviations from the intervention. |
| Page 14: "And finally, one of the answers used no grammatical terminology whatsoever, but instead represented an answer based on everyday language and experiences.": How much would students score in this case? say it (like you did before), even if it is in the table. | Done. |
| Page 16: "Additional Bonferroni post hoc tests": Additional Bonferroni adjusted post hoc tests | Corrected |
| Pages 18-20: Quantitative analysis: I suggest you try to link the tests you have performed with the research questions you are trying to answer. It may be easier to follow, especially for non-experts. | We have started this section with our research questions in the new version – we think this makes it easier to follow. The core variables can then be linked to the RQ’s easier. |
| Page 18: "At measurement moment 3": 3 or 2? | No, this should be moment 3. After all, both groups will have had the treatment at that stage. They will not all have had the treatment at M2. |
| Page 24: "From Table 4, it can be inferred that [...]": could you please guide your reader in the reading of the table? What specifically supports your claims? is it an increase/reduction in the scores? Say it. You do it properly in other parts. | We agree that this could have been explained clearer. We have therefore revised the text as follows: From Table 4, it can be inferred that for the Understanding Scores and the No Concept Scores there is an effect of the intervention (for both Model 2 outperforms Model 1, which is reflected in the higher elpd values for Model 2 as compared to Model 1), although there is no effect for the Blind Scores (more complex models do not outperform Model 1, reflected in Model 1 having the highest elpd value). |
| Page 24: Table 5: Are these values in the natural log?  It would be very interesting to present some proposed verbal equivalents for likelihood ratios, like in a table. It would help the reading and the understanding of your results considerably. Not everyone is familiarized with the Bayesian analysis and the way the outcomes are reported. It would be much easier to grasp Table 5, for instance. | The values in Table 5 are expressed on the scale of the original variables. No values in the log scale; we are not sure why the reviewer suggests this might be the case. For example: Intercept Occ1 for Understanding: the value 10.149 represents a point estimate of the expected score at the first moment. The 95% HDI means that the 95% most likely value for the score at time 1 is somewhere between 9.7 and 10.6 points. We hope this clarifies the Table. Since we have followed standard reporting procedures for Bayesian analyses, we have kept our initial table intact, although we have made it easier to read in the new version. |
| Page 26: Figure 3: Although it may seem repetitive, you need to remind your readers what group 1 and group 2 are. It is easy to forget. | We agree, and have now done so. |
| Table 8 and 9: You need to separate your rows better. It is very confusing the way it is. We need to make an effort to understand when things start or end in the "Definition of subcode" column. | We agree with this too; the table should now be much more readable. |
| The title of Table 9 is out of the table. | True, but MS Word does not seem to agree with us if we are trying to move it to the Table. We will make sure it will be correctly formatted after acceptance. |
| Page 38: Although the researchers have a good sense of the study limitations, it is odd that you start the discussion by first stating it. Why don't you start by discussing your main findings instead? I suggest you leave the study limitations for last, before the conclusions*. | We have now switched these things around as suggested – this seems to have improved the general flow of the manuscript. Thank you for this suggestion! |
| In the introduction (page 12, second paragraph), you report that one of the main differences between your study and the one conducted by [Van Rijt et al.] was that rather than focusing on four different linguistic  metaconcepts, the intervention focused on just one: valency. I thought this difference should be highlighted and better exploited in the discussion, especially considering that you did not find a negative side-effect in your study, but they did. One would expect that having a larger number of linguistic concepts/terminologies to handle would impact the students' performance in general. You refer to it in your introduction when saying that "students would have the opportunity to process the newly taught information more effectively, limiting the  amount of new linguistic terminology", however, you do not explore this in your discussion, you only suggest (last paragraph of page 39) that this may be because in [Van Rijt et al.] students were prompted to writing reasonings. | This is indeed a very valid point. We have elaborated on this in the discussion as follows (p. 40):  An alternative explanation might be that, as stated in the introduction, the current intervention only targeted one metaconcept (valency), while the Van Rijt et al. [56] intervention targeted four metaconcepts. It would seem likely that the chances of blind (meta)concept use increase when there are more metaconcepts that have to be learned in the first place. Differences in conceptual content covered in the interventions could thus account for differences in blind concept use between the current study and Van Rijt et al.’s [56]. |
| Page 41: *No conclusions section? I find it interesting to provide your reader a summary of your primary outcomes. | We have now added a brief conclusions section 😊 |
| You have also reported several textual issues, for which we are grateful. | We have followed up on *every single issue*, except for the comma before ‘and’ here: ‘0.2, 0.4 and 0.6 -> 0.2, 0.4, and 0.6’, because according to several linguistic sources, such comma’s are debatable (some even recommend not putting one before ‘and’ in cases such as these). |
| **R2** |  |
| I'm already familiar with the research coming from this group, and I think it's one of the best research programs in pedagogical linguistics at the moment. The present paper presents original and new findings, based on previous studies. Importantly, the main finding (at least for me) is that students' blind concept use is actually an integral part of a gradual understanding of grammatical concepts. This is extremely interesting and also relevant for other domains of teaching and learning. Given my positive evaluation of this manuscript (and of the underlying research program as a whole), I only have three very minor comments for the authors: | Thank you, we are very honored to receive such an assessment. We have happily considered your suggestions. |
| 1) Students start out by using strategies like 'name dropping' etc. before they are able to use grammatical concepts appropriately. Is there a general sequential model for this in the educational sciences/in learning theory? The 'fake-it-till-you-make-it' strategy seems to be a common developmental step in other domains too, so I'm wondering whether the authors could clarify this aspect of their findings even further, making their research even more relevant for a journal like PLOS ONE. (Note though that this strategy cannot really be applied in other subjects such as math. Hudson 2020 points out some parallels and differences between linguistics and other school subjects, and I think this might be a new point in the context of his discussion.) | Yes, this is an interesting point. We are not aware of any sequential models for such conceptual learning in education, although we see interesting links with Cummins’ work [91], so we have related this finding to findings from other fields as follows (p. 41):  This struggle between everyday concepts and subject-specific concepts has also been observed in science education, where students have, for example, been found to struggle with learning about the scientific concept of ‘energy’ [90], which also has an everyday equivalent. A similar observation has been made in students’ use of historical concepts in history education. Not only do students typically use historical concepts in a very limited way when reasoning about history, but according to Havekes (p. 69), they also ‘apply everyday discourse to describe a historical concept, such as ‘changes in the church’ instead of ‘reformation’’[66]. These examples suggest that an integral part of conceptual learning might be that students have to learn how to deal with tensions between everyday concepts on the one hand and scientific concepts that they are expected to master in the context of a school subject on the other (see also the distinction between Basic Interpersonal Communicative Skills (BICS) and Cognitive Academic Language Proficiency (CALP)[91]). In this sense, the current study shows that there might be parallels between conceptual learning in linguistics education and conceptual learning in other fields. We leave further exploration of this matter open for future research. |
| ) The authors correctly point out the limitations of their study, one of them being that  "there is no data to shed more light on students’ reasoning (for example from think aloud protocols), that could deepen our understanding of students’ understanding both within and outside of the intervention."  => Could the authors point out ways to (systematically) implement such a qualitative component in future studies? It's always hard to analyze such protocols properly because researchers could basically pick out any aspect of it that might fit their findings best. How could such protocols be properly coded and analyzed according to the authors? | Yes, this is always hard. We have not incorporated this into our initial manuscript because we felt that such recommendations might make the study less focused in its discussion, but upon further reflection, we have decided to add the following (p. 43):  Such data is only useful if think aloud protocols are applied in a way that avoids the pitfall of cherry picking. One way to achieve this is to randomize which students participate in the protocol; another is to pre-structure the protocol, focusing only on a few pre-determined variables, so that all of the data can be coded, rather than a few pieces of data that might match the researchers’ needs. A final recommendation regarding the role of thinking aloud protocols is to use them retrospectively (rather than simultaneously to the task), as this increases their validity [93]. |
| “For example, some students used the concept of role in an everyday sense rather than in a metalinguistic sense, indicating that they have not yet fully grasped the grammatical concept yet. Their use of the word role may therefore be seen as an indication of a limited understanding, although, encouragingly, it may also mean that students are on a conceptual journey, a part of which is to struggle with new terminology and concepts."  => I was wondering whether this also holds for the term "grammar" itself. Our everyday concept of grammar is fundamentally different from the one in linguistics (prescriptive vs. descriptive, social norms vs. cognitive capacity etc.); see Döring (2020) for a recent paper on this. It would be nice if the authors could add this more general aspect to their final discussion. | We have now added a comment about this (p. 41):   Another case in point would be that students experience some conceptual confusion as a result of the traditional ways in which grammar is usually taught. Students might not be accustomed to reason within the context of grammar instruction, which is usually mostly concerned with superficial parsing exercises [34, 35]. This pertains to the meaning of the very concept of grammar in this study, as the everyday use of this concept is rather different from the linguistic concept of grammar (with the former being mostly related to prescriptivism and language norms, and the latter being mostly associated with descriptivism and cognitive capacity). Students have been shown to hold views on grammar that are more related to the everyday meaning than to the linguistic meaning of the word [91-92].  In doing so, we have included the suggested reference to Döring’s work, as well as a reference to Nygard & Broseth (2021). |
